# Adjusting health expenditure for military spending and interest payment: Israel and the OECD countries

**DOI:** 10.1186/2045-4015-2-5

**Published:** 2013-02-20

**Authors:** Amir Shmueli, Avi Israeli

**Affiliations:** 1 The Hebrew University–Hadassah School of Public Health, POB 12272, Jerusalem, 91120, Israel

**Keywords:** Health spending, International comparisons, Israel, Military spending, Interest payment

## Abstract

**Background:**

Compared to OECD countries, Israel has a remarkably low percentage of GDP and of government expenditure spent on health, which are not reflected in worse national outcomes. Israel is also characterized by a relatively high share of GDP spent on security expenses and payment of public debt.

**Objectives:**

To determine to what extent differences between Israel and the OECD countries in security expenses and payment of the public debt might account for the gaps in the percentage of GDP and of government expenditures spent on health.

**Methods:**

We compare the percentages of GDP and of government expenditures spent on health in the OECD countries with the respective percentages when using *primary civilian* GDP and government expenditures (i.e., when security expenses and interest payment are deducted). We compared Israel with the OECD average and examined the ranking of the OECD countries under the two measures over time.

**Results:**

While as a percentage of GDP, the national expenditure on health in Israel was well below the average of the OECD countries, as a percentage of primary civilian GDP it was above the average until 2003 and below the average thereafter. When the OECD countries were ranked according to decreasing percent of GDP and of government expenditure spent on health, adjusting for security and debt payment expenditures changed the Israeli rank from 23rd to 17th and from 27th to 25th, respectively.

**Conclusions:**

Adjusting for security expenditures and interest payment, Israel's low spending on health as a percentage of GDP and as a percentage of government's spending increases and is closer to the OECD average. Further analysis should explore the effect of additional population and macroeconomic differences on the remaining gaps.

## Background

The percentage of GDP (Gross Domestic Product) spent on health has traditionally been used in evaluating the weight of the health sector within the economy. Similarly, the percentage of the government’s expenditures spent on health has been used in evaluating the weight of the health sector to the government within the government’s activities. These evaluations are used to explore how a country’s health sector has evolved over time or to compare the health sector of different countries at a given time.

International comparisons of these measures have several shortcomings, some of which have been discussed in the professional literature in Israel [1-4] and elsewhere [5,6]. In general, these comparisons do not take into account important determinants of the national expenditures on health: the characteristics of the populations (e.g., age, morbidity, lifestyle, diet, genetic diseases), the characteristics of the health systems (e.g., service availability, insurance coverage, incentives), macroeconomic characteristics, and the unique national characteristics of specific nations. Despite these problems, international comparisons of the percent of GDP and of government expenditure spent on health continue to be used intensively.

In this article we focus on two of the most basic national characteristics: a country’s security needs and preferences regarding its military expenditure, and the payment of its public debt. Prof. Stanley Fisher, Governor of the Bank of Israel, stated (our translation) in a TV interview on August 10, 2012: "The main responsibility of any country is to keep its own security." Similarly, payment of the public debt is also considered a high priority because of its importance in preserving any country's standing and reputation and also its credit terms in the international capital markets. We therefore assume that the allocation of national and governmental resources to the various uses – at least in Israel, the focus of this comparison – is determined sequentially: the amount of resources allocated to security and to paying the debt are determined first according to the country's needs and preferences, and funding for all the other national needs is taken from the remaining*, primary civilian*, resources. In other words, we argue that funding for health expenditure is taken from the country’s *primary civilian resources* (i.e., the resources after security expenses and payments of the debt are deducted) and not from the *total* amount of resources. Dahan and Hazan [2] make a similar assumption when analyzing public and social expenditure in Israel and OECD countries. A confirmation of the sequential nature of the allocation process in Israel could be viewed in the 2011–2012 budget discussions: the Ministry of Finance insisted on cutting the security budget in order to make possible an increase in government expenditure on primary civilian uses such as education, welfare, and health. On July 16, 2010, Prime Minister Netanyahu decided to cut NIS 2.7 b out of the security budget and to allocate these funds to primary civilian uses.

Obviously, since Israel’s security and military *needs* are much higher than in the OECD countries, the share of (total) resources allocated to military expenditure is much higher than in the OECD countries, and the difference between total and primary civilian resources is significant. When the country's security *needs* and the interest payments are relatively low, the difference between the amount of total resources and the amount of primary civilian resources will obviously be small. For illustration, in 2008, interest payments out of GDP were 3.6% in Israel, 2.3% in Germany, 0% in Canada, 2.7% in France, 1.9% in the UK, and 1.8% in the U.S. The OECD average was 1.1%. The differences are much more pronounced in military spending. Israel spent 7% of its GDP on security and military, while the OECD average was 1.8%. The OECD range included the U.S. with 4.3%, the UK and France with 2.5%, and Japan with 1%.

Consequently, we argue that when comparing health expenditure between Israel and the OECD countries, the health expenditure should be evaluated as a percentage of the *primary civilian* resources and not the total resources.

## Methods

In this paper we compare the share of GDP and "primary civilian GDP" of national health spending as well as the share of government expenditure on health in total, and in primary civilian government expenditure in Israel and in the OECD countries. Both in Israel and in the OECD countries, "government" stands for the "general government", including social security and other governmental agencies (the expenditure of the "general government" is sometimes referred to as "public" expenditure).

We denote the percentage of total resources spent on health with the letter ‘r’ and the percentage of the government (and national) total expenditure spent on security and payment of the debt with the letter ‘s’. The primary civilian resources are (100-s)% of the total resources; the percentage of the primary civilian resources spent on health is therefore v=100r/(100-s). For example, if the percentage of total resources spent on health is 10% (r=10) and the percentage spent on security and the payment of the debt is 5% (s=5), then the primary civilian resources are 95% of the total resources and health expenditure is 10.5% of the primary civilian resources. Using the figures presented above, the primary civilian GDP in 2008 was 89.4% of total GDP in Israel, and 97.1% on average in the OECD.

Health spending as percent of GDP data was taken from the OECD HEALTH DATA 2010. Military expenditures and net interest payment as percent of GDP were taken from the Economic Outlook No 88 – December 2010 – Annual Projections for OECD Countries. We covered the period 1993–2008. Government health spending as a percentage of total government spending for the period 1995–2008 was calculated as the ratio of government health spending as a percentage of GDP (retrieved from OECD HEALTH DATA 2010) to government total spending as a percent of GDP (retrieved from Economic Outlook No 88 – December 2010 – Annual Projections for OECD Countries).

For some of the 34 OECD countries not all of the information existed for all relevant years. The information for five of the countries (Chile, Mexico, Turkey, Greece, and Estonia) was incomplete (mostly regarding debt payment) for a large proportion of the years and therefore these countries were omitted from the analysis. The number of countries used for each analysis varied between years and sequences, according to the availability of data.

We used two ways to examine the effect of using primary civilian resources instead of total resources for calculating the share of health spending. First, we calculated for each year the non-weighted mean (as is common in many analyses of OECD data) of each relevant sequence over all the OECD countries (including Israel) with existing values and compared the results with the Israeli values. Second, in each year we ranked the OECD countries according to the share of health spending of total resources available and according to the share of primary civilian resources, and compared the two rankings.

## Results

**Figure 1:**
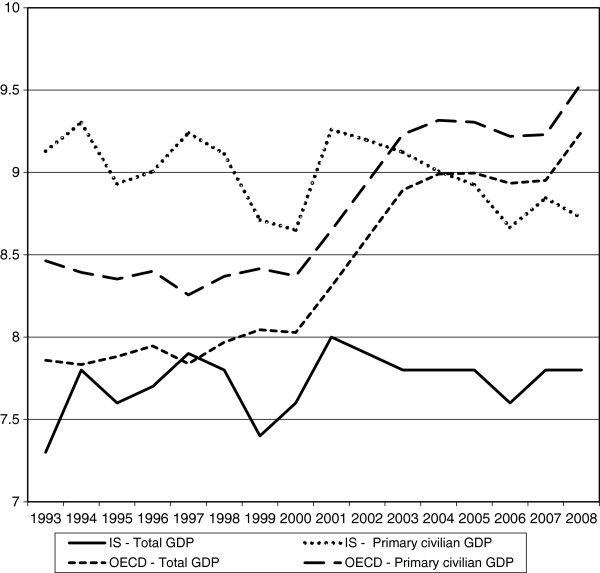
The percentage of total and primary civilian GDP spent on health – Israel and the OECD average.

Figure 1 shows the percentage of GDP spent on health (‘r’) and the percentage of primary civilian GDP spent on health (‘v’) over 1993–2008 in Israel and on average in the OECD countries. From Figure 1 it is apparent that, on average, from 1998 the percentage of the GDP that OECD countries spent on health rose while in Israel it was practically constant. As a result, by 2008 this percentage was 7.7% in Israel while the OECD average was 9.3%. It should be noted that this measure is affected by the level of national expenditure on health, and also by the size of the GDP and its fluctuations along the business cycles. 

When we evaluate the percentage of the primary civilian GDP spent on health we get a completely different picture. The percentage of primary civilian GDP spent on health in Israel was about half a percent (about 8.5% vs. 9%) *above* the average of the OECD countries until 2003 and below it thereafter. In 2008, the percentage of the primary civilian GDP allocated to health was 8.7% in Israel while the average of the OECD countries was 9.6%. This difference is about half the difference between Israel and the OECD average in 2008 when total GDP is used in the evaluation.

**Figure 2:**
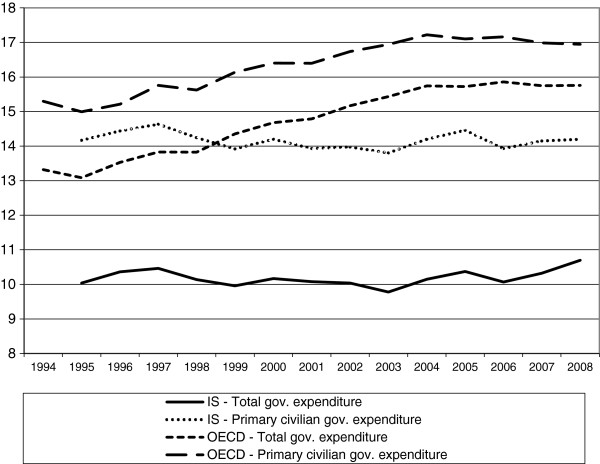
The percentage of the government’s total and primary civilian expenditures spent on health – Israel and the OECD average.

Figure 2 shows the percentage of the general government’s total expenditures spent on health (‘r’) and the percentage of the general government’s primary civilian expenditures spent on health (‘v’) during 1995–2008 in Israel and on average in the OECD countries. While in Israel the percentage of the government’s total expenditures spent on health was about 10% throughout the period, the OECD average rose from about 13% to 16%. When we evaluated health expenditure as a percentage of the government’s *primary civilian* expenditures, we found that the Israeli rate was constant at about 14%. The impact of using the adjusted evaluations was less dramatic on the average of the OECD countries. The percentage of the government’s *primary civilian expenditures* spent on health on average in the OECD countries remained above the percentage in Israel, but the difference dropped from about 6 percentage points to only 2.5 percentage points. 

The graphs for Israel in Figure 2 are practically parallel, which is an outcome of the fact that the percentage of the government’s expenditure spent on health was constant both when the government’s total expenditures was used and when the government’s primary civilian expenditures was used. With regard to the average of the OECD countries, it seems that until 2004 expenditure on security and paying the debt declined and was fairly stable thereafter. The difference between total government expenditure and primary civilian government expenditure was significantly larger in Israel than the OECD average throughout the period.

**Figure 3:**
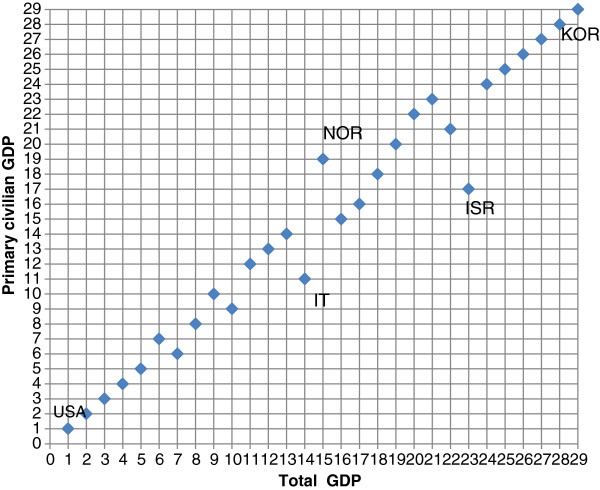
Ranking OECD countries by health spending as % of GDP – 2006.

Figure 3 presents the ranking (from high (1) to low (29)) of the OECD countries by health spending as percentage of GDP in 2006. The horizontal (x) axis indicates the ranking when the total GDP is used, and the vertical (y) axis indicates the ranking when the primary civilian GDP is used. For 11 countries, the 6 countries with the lowest share of total GDP spent on health (with Korea having the lowest share of 6.1%) and the 5 countries with the highest share (led by the U.S. with a share of 15.5%), the ranking did not change when using primary civilian instead of total - GDP. Israel advanced from 23rd place (7.6%) to 17th place (8.7%). It is interesting to note that Italy, a country with relatively high interest payments, advanced from 14th place (9%) to 11th (9.6%). Norway, on the other hand, with negative net interest payment, moved from 15th place to 19th, although the share dropped merely from 8.6% to 8.5%. The remaining countries moved up or down 1 or 2 places. 

**Figure 4:**
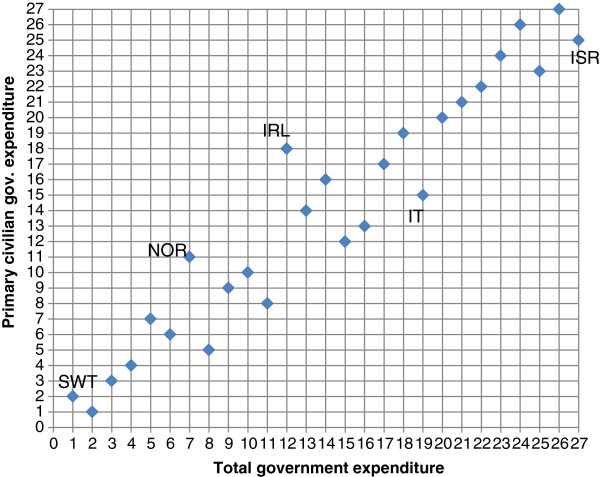
Ranking OECD countries by government health spending as % of government spending – 2008.

Figure 4 presents a similar analysis but with the general government spending on health as a percentage of government spending. Israel, the country with the lowest share (9.8%) of health spending out of total government spending (27th place on the horizontal axis) climbed to the 25th place (12.7%) when primary civilian government expenditure is used. Italy also moved from 19th place (14.4%) to 15th place (16.6%). On the other hand, Ireland moved from 12th place (15.8%) to 18th place (16.5%), and Norway – from 7th place (17.8%) to 11th place (17%). Switzerland, the country with the highest share (1st place on the horizontal axis) of 19.7% moved to the 2nd place (20.5%) when using primary civilian government expenditures instead of total expenditures. 

## Discussion and Conclusions

One of the popular methods of comparing the importance attributed to the health sector between different countries is to compare the percentage of the total GDP and the government’s total expenditure spent on health in the different countries [5,6]. According to these comparisons, Israel was quite far below the OECD average during the last decade. Furthermore, in 2006 Israel was ranked 23rd out of 29 in the share of health spending out of GDP (1st place signifies the highest share), and in 2008, it was ranked 27th (lowest) in the share of government spending on health.

There are several possible explanations for these differences, including differences in population age, the structure of the health systems, and other macroeconomic characteristics. The low share of the Israeli government spending on health might be partially attributed to dental care being financed privately out-of-pocket in Israel, and to the lower taxes collected in Israel due to the lower GDP per capita relative to most OECD countries.

We examined one particular measurement-related explanation for the differences between Israel and the OECD countries – the different national needs faced by countries allocating their national resources. Two such needs, which are common for most countries – and for Israel in particular – are security and payment of the public debt. And indeed, when the unique security constraints in Israel and the relatively high payment of its debt are taken into account, the percentage of *primary civilian* GDP spent on health in Israel is much closer to the average of the OECD countries and was even above it until 2003. In 2006, this adjustment moved Israel from 23rd place out of 29 to 17th.

One of the claims against the Israeli government is that it has been encouraging the replacement of public spending on health with spending by the private sector. This could have many implications regarding fairness and equality, the discussion of which goes beyond the scope of this paper. Because security and the payment of the public debt are government expenses, we focused on the percentage of the government’s primary civilian expenditures spent on health. The Israeli percentage is still lower than the average of the OECD countries throughout the period. However, it is much closer to the average than when the percentage of the government’s total expenditures spent on health is used. Israel's rank moved from 27th place (the lowest) among 27 OECD countries to 25th.

### Conclusions

These findings are significant in two ways. First, we believe that using primary civilian instead of total GDP and government expenditure better reflects the priority given to health within the allocation of national and government resources. This is particularly true when Israel is compared to other countries, and such adjustment leads to more accurate and sensible conclusions. On a larger scale, we believe that more attention should be given to the issue of national needs and priorities in international comparisons of expenditures and uses of the GDP and government consumption. Second, they show that even when we deduct Israel’s top national priorities, security and payment of debts, from the national and public expenditures, the percentage of primary civilian GDP spent on health has been declining since 2003 and is below the average of the OECD countries. The percentage of the government’s primary civilian expenditures on health is also lower than the average of the OECD countries, and Israel has one of the lowest shares among these countries. While there are no "gold standards" that can be used to evaluate the percent of GDP or of government expenditure spent on health [7,8], further analysis is needed in order to better understand the sources of these differences and their implications on the Israeli population health.

## Competing interests

The authors declare that there are no competing interests.

## Authors’ contributions

AS and AI designed the study and wrote the paper. AS did the empirical analysis. Both authors read and approved the final manuscript.

## Authors’ information

Amir Shmueli is a professor of health economics at The Hebrew University–Hadassah School of Public Health.

Avi Israeli is the Dr. Julien Rozan Professor of The Hebrew University–Hadassah School of Public Health and the Ministry of Health at the Hebrew University – Hadassah Faculty of Medicine; Director of the Department of Health Policy, Health Care Management and Health Economics, Hebrew University – Hadassah Braun School of Public Health & Community Medicine; Chief Scientist of the Ministry of Health; and co-editor of the IJHPR
